# Validation of the NoSAS score for screening sleep-disordered breathing: A sleep clinic-based study in Turkey

**DOI:** 10.3906/sag-2002-54

**Published:** 2021-02-26

**Authors:** Burcu OKTAY ARSLAN, Zeynep Zeren UÇAR, Özgür BATUM, Mehmet Nurullah ORMAN

**Affiliations:** 1 Department of Sleep Disorders, Dr. Suat Seren Chest Diseases and Chest Surgery Training and Research Hospital, University of Health Science, İzmir Turkey; 2 Department of Chest Diseases, Dr. Suat Seren Chest Diseases and Chest Surgery Training and Research Hospital, University of Health Science, İzmir Turkey; 3 Department of Biostatistics, Faculty of Medicine, Ege University, İzmir Turkey

**Keywords:** NoSAS score, sleep-disordered breathing, STOP-Bang, Berlin questionnaire

## Abstract

**Background/aim:**

The NoSAS score is a new tool for the identification of high-risk patients for sleep-disordered breathing (SDB). The aim of this study was to validate the NoSAS score in a sleep clinical population in Turkey and compare its performance with the Epworth Sleepiness Scale (ESS), STOP-Bang, and Berlin questionnaires for high-risk SDB.

**Materials and methods:**

This was a retrospective study. Patients who had a full-night PSG examination between 01.03.2017 and 01.01.2018 at the sleep center of our hospital were included in the study. Demographic characteristics, anthropometrics measurements, ESS, STOP-Bang, and Berlin scores were collected from the existing data of the patients. The NoSAS score was subsequently calculated based on available data. Predictive parameters for each screening questionnaires were calculated to compare the discriminative power of those for high-risk SDB.

**Results:**

A total of 450 patients were included in the study. The sensitivity, specificity, PPV, and NPV of the NoSAS score were 81%, 51.2%, 88.2%, and 37.5% for an AHI (apnea–hypopnea index) ≥ 5 event/h and 84.5%, 38.2%, 66%, and 63.4% for an AHI ≥ 15 event/h, respectively. AUC percentages for the NoSAS score, STOP-Bang questionnaire, Berlin questionnaire, and ESS were 0.740, 0.737, 0.626, and 0.571 for an AHI ≥ 5 events/h and 0.715, 0.704, 0.574, and 0.621 for an AHI ≥ 30 events/h. The NoSAS score had a false negative rate of 2.9% for severe SDB.

**Conclusion:**

The NoSAS score had a good degree of differentiation for SDB and can be used as an easily applicable, subjective, and effective screening tool in a sleep clinical population in Turkey. Not only in moderate to severe SDB but also in mild SDB, the NoSAS score performed better than the other 3 screening tools.

## 1. Introduction

Sleep-disordered breathing (SDB) is a highly prevalent and grossly underrecognized disease that is characterized by repetitive collapse of the upper airway during sleep. It has been suggested that 34% of men aged 30–70 and 17% of women aged 30–70 have SDB [1]. It has been estimated that up to 80% of individuals with moderate to severe SDB may remain undiagnosed and, furthermore, untreated [2]. Patients with untreated sleep-disordered breathing are at increased risk of hypertension, stroke, heart failure, diabetes, car accidents, and depression [3]. Currently, the gold standard for diagnosing SDB is a full-night polysomnography (PSG). However, the procedure is time-consuming, expensive, complex, and relatively inaccessible; it also requires technical personnel. Long waiting periods for sleep studies are still an important problem for the diagnosis of OSA, even in developed countries [4]. Screening questionnaires are simple, easy to use, and low-cost tools that can be used to prioritize the patients eligible for PSG. Different clinical questionnaires have been previously proposed as screening tools for SDB [5]. The STOP-Bang questionnaire, Berlin questionnaire, and Epworth Sleepiness Scale (ESS) are the most commonly used screening tools for SDB [6]. ESS was originally designed to evaluate daytime sleepiness [7]. The STOP-Bang and Berlin questionnaires were developed using less sensitive technology than those currently used. Considering these technical differences and their effects on the diagnosis and perceived prevalence of SDB, Marti-Soler et al. developed a new screening tool for SDB in a cohort study of 2121 subjects in a Swiss population (HypnoLaus cohort) using current standards as a reference. The NoSAS score was also validated by another cohort in Brazil (EPISONO) that included 1042 subjects to ensure its reliability in different populations. The results of this study revealed that the NoSAS score can be used as simple, efficient, and easy to implement score to identify individuals at risk of SDB [8]. However, different clinical populations may present different results. As a result, the effectiveness of the NoSAS score in different clinical populations should be evaluated.

The purpose of the current study was to validate the NoSAS score in a sleep clinical population in Turkey and compare its performance with the STOP-Bang and Berlin questionnaires and with the Epworth Sleepiness Scale to predict high-risk patients for SDB.

## 2.Material and methods

### 2.1. Subjects

This study was a retrospective analysis of patients who had already had a full-night PSG examination between 01.03.2017 and 01.01.2018 at the sleep center of the University of Health Science, Dr. Suat Seren Training and Research Hospital. All patients were suspected of having SDB. The criteria for inclusion were as follows: (1) over 18 years of age; (2) no previous diagnosis and treatment of SDB; (3) completed anthropometric data regarding ESS, STOP-Bang, and Berlin Questionnaires in a sleep laboratory; and (4) a sleep efficacy ≥60%. Those who did not meet these criteria were excluded from the study. Additionally, patients who had an active psychiatric disorder, a history of brain tumors, epilepsy, or had used benzodiazepine were also excluded from the study.

In our study, basic demographics (e.g., age and sex), anthropometric measurements (height, weight, BMI, and neck circumference), ESS, STOP-Bang questionnaire, Berlin questionnaire, and PSG parameters (apnea–hypopnea index (AHI), oxygen desaturation index (ODI), and sleep efficacy) were collected from the existing data of the patients. The comorbidities of all patients were also recorded. The NoSAS score was obtained using the existing data of the patients. 

### 2.2. Screening questionnaires

The ESS is an eight-item questionnaire; it uses a four-point Likert response format (0–3), and the score ranges from 0 to 24. An ESS score of ≥10 indicates excessive daytime sleepiness and high risk for OSA [7]. 

The Berlin questionnaire incorporates 11 questions organized into 3 categories. The first category includes 5 questions about snoring, the 2nd category includes 3 questions about daytime sleepiness and fatigue, and the last category includes information about the history of hypertension and body mass index (BMI). The overall Berlin score was determined from the responses to these 3 categories: the 1st and 2nd categories are considered positive if the responses indicate frequent symptoms (>3–4 times/week) on 2 or more questionnaire items, and the 3rd category is considered positive if there is a history of hypertension or a BMI >30 kg/m2. Patients who had a positive score on 2 or more categories were classified as being at high-risk for OSA [9].

The STOP-Bang questionnaire includes 8 dichotomous (yes/no) questions, 4 of which are subjective (STOP: snoring, tiredness, observed apnea, and high blood pressure) and 4 of which are demographic (Bang: BMI >35 kg/m2, age >50 years, neck circumference >40 cm, male sex). The total score ranges from 0–8. Answering ‘yes’ to 3 or more question places the patient at high risk for SDB [10]. We used a valid Turkish language version of the 3 questionnaires [11–13] .

The NoSAS score is a new screening tool. This score, which ranges from 0–17, allocates 4 points for having a neck circumference of more than 40 cm, 3 points for having a BMI of 25 kg/m² to less than 30 kg/m² or 5 points for having a BMI of 30 kg/m² or more, 2 points for snoring, 4 points for being older than 55 years of age, and 2 points for being male. An NoSAS score of 8 or higher is indicative of being at high risk for SDB [8]. 

### 2.3. In-laboratory polysomnography

The diagnosis of OSA was made using an in-lab polysomnographic examination. Electroencephalography, electrooculography, electromyography of the chin and the leg (anterior tibialis), electrocardiography, oxygen saturation (from fingertips), respiratory effort (thoracic, abdominal) and air flow (nasal pressure transducer and oronasal thermistor), body position, and tracheal microphone were recorded with the Comet Grass Telefactor version 4.5.3 (Comet Group, Flamatt, Switzerland). Polysomnography recordings were analyzed by a physician experienced in sleep disorders using TWin EEG/PSG Software. Scoring of the sleep and respiratory events were done according to the criteria of the AASM manual version 2.3 [14]. The AHI was defined as the number of apnea and hypopnea events per hour. The diagnosis of OSA was defined by AHI. The severity of SDB was categorized as follows: mild (5 ≤ AHI < 15 events/h), moderate (15 ≤ AHI < 30 events/h), and severe (AHI ≥ 30 events/h).

### 2.4. Statistical analysis

Statistical analyses were performed using IBM-SPSS version 25.0 (IBM Corp., Armonk, NY, USA). The demographic data was presented with descriptive statistics. Numerical data were given as the mean ± standard deviation (SD), and frequency data were given as number and percentage (%). The concordance of numerical variables with normal distribution was evaluated with the Shapiro–Wilk test. For normal distributions, a Student’s t-test was used to compare the 2 groups. Cross tabulation was used for categorical data and chi-square analysis was performed. PSG was considered as the gold standard and the sensitivity, specificity, positive predictive values (PPV), and negative predictive values (NPV) of the ESS, Berlin, and STOP-Bang questionnaires, along with the NoSAS score according to specific cut-off values, were calculated. Receiver-operating characteristic (ROC) curves were constructed to assess the ESS, Berlin, STOP-Bang questionnaires, and the NoSAS score regarding their likelihood to predict high-risk for OSA. Correlation between the questionnaires was analyzed by Pearson’s correlation coefficient. All tests were two-sided and statistical significance was assumed when P < 0.05. 

All procedures performed in studies involving human participants were in accordance with the ethical standards of the institutional and/or national research committee and with the 1964 Helsinki Declaration and its later amendments or comparable ethical standards. The study was approved by the local research ethics committee (23.10.2018, Number: 11688). 

## 3. Results

**Figure 1:**
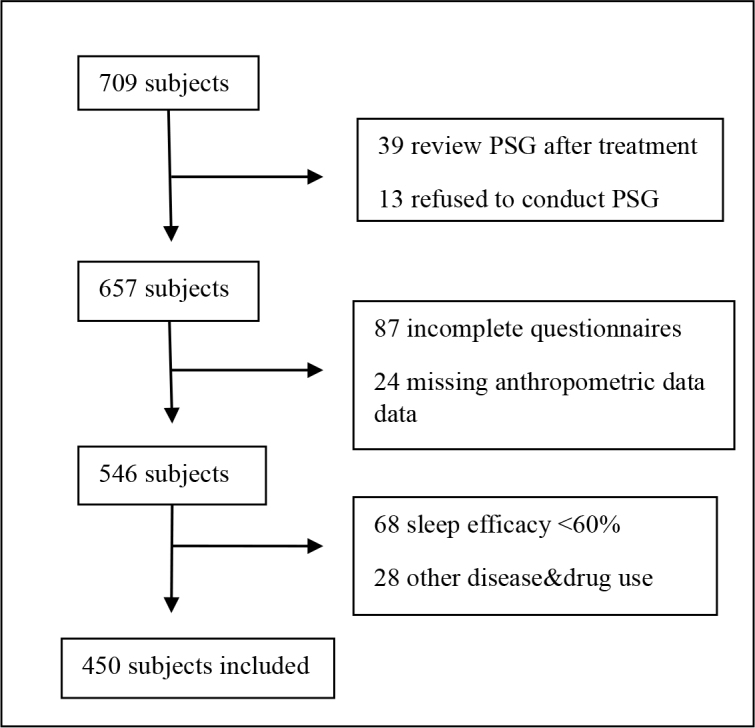
Flow diagram. PSG: Polysomnography.

A total of 450 patients were included in the study (Figure 1). Approximately 66.7% of the study population was male and 33.3% females. The mean age of the subjects was 50.6 ± 11.3. Demographic and anthropometric characteristics of the study population are presented in Table 1. According to the PSG results: 104 patients (23.1%) had mild SDB, 122 patients (26.6%) had moderate SDB, and 142 patients (31.5%) had severe SDB. 

**Table 1:** Baseline characteristics of the study population (n: 450).

	AHI < 5 events/h	AHI ≥ 5 events/h	P value
Number (%)	82 (18.2)	368 (81.7)	
Male sex, N (%)	38 (46.3)	262 (87.3)	<0.001
Age, year	44.3 ± 11.5	51.3 ± 11.3	<0.001
BMI, kg/m2	29.0 ± 5.2	32.1 ± 5.9	<0.001
Neck circumference, cm	39.2 ± 2.95	41.5 ± 3.1	<0.001
Smoker, N (%)	38 (46.3)	153 (41.6)	0.430
Snoring, N (%)	73 (89)	353 (95,9)	0.012
Witnessed apnea ,N (%)	56 (68.3)	309 (84)	0.001
Daytime sleepiness, N (%)	71 (86.6)	320 (87)	0.928
NoSAS	7.9 ± 4.0	11.4 ± 3.3	<0.001
STOP-Bang	3.9 ± 1.4	5.2 ± 1.3	<0.001
Berlin	2.0 ± 0.8	2.4 ± 0.6	<0.001
ESS	8.3 ± 5.4	9.9 ± 6.1	0.031
Hypertension, N (%)	21 (25.6)	142 (38.6)	0.027
Diabetes Mellitus, N (%)	12 (14.6)	97 (38.6)	0.025

Data is depicted as mean ± SD or number (percentage).

The effectiveness of the NoSAS score as a screening tool for SDB was evaluated using the different cut off points of AHI. Table 2 shows the predictive parameters of the NoSAS score, STOP-Bang questionnaire, and Berlin questionnaire for AHI cutoffs of 5, 10, 15, 20, 25, and 30 events/h. The sensitivity of the NoSAS score increases as the AHI increases. For an AHI ≥ 5, the sensitivity, specificity, and PPV and NPV of the NoSAS score were 81%, 51.2%, 88.2%, and 37.5%, respectively. On the basis of its ability to discriminate subjects with clinically significant SDB (i.e. an AHI of ≥15 events/h), the sensitivity, specificity, and PPV and NPV of the NoSAS score was 84.5%, 38.2%, 66%, and 63.4%, respectively. The STOP-Bang questionnaire had the highest sensitivity for all AHI cut off points but also had the lowest specificity. The Berlin questionnaire demonstrated similar results to the STOP-Bang questionnaire. For an AHI of ≥15 events/h, the sensitivity, specificity, and PPV and NPV of the ESS was 53.8%, 59.1%, 65.1%, and 47.4%, respectively.

**Table 2:** Predictive parameters of the NoSAS score and STOP-Bang and Berlin questionnaires for AHI cut offs of 5, 10, 15, 20, 25, and 30 events/h.

	AHI≥5	AHI≥10	AHI≥15	AHI≥20	AHI≥25	AHI≥30
NoSAS score						
Sensitivity (%)	81	83	84.5	86	87.3	90.8
Specificity (%)	51.2	42.8	38.2	35.5	33.1	32.1
PPV(%)	88.2	76.6	66	56.5	46.7	38.2
NPV (%)	37.5	52.7	63.4	72.3	79.5	88.4
STOP-Bang						
Sensitivity (%)	97.8	97.8	98.5	98.6	98.9	98.6
Specificity (%)	15.9	10.1	9.1	7.9	7.1	6.2
PPV (%)	83.9	71.1	60.6	51	41.7	32.6
NPV (%)	61.9	66.7	81	85.7	90.5	90.5
Berlin						
Sensitivity (%)	89.9	89.4	88.6	90.1	91.2	93.7
Specificity (%)	26.8	18.8	15.6	16.2	16	16.2
PPV (%)	84.7	71.4	59.8	51.2	42.2	34
NPV (%)	37.3	44.1	49.2	62.7	72.9	84.7

AHI: apnea–hypopnea index; PPV: positive predictive value; NPV: negative predictive value.

**Figure 2:**
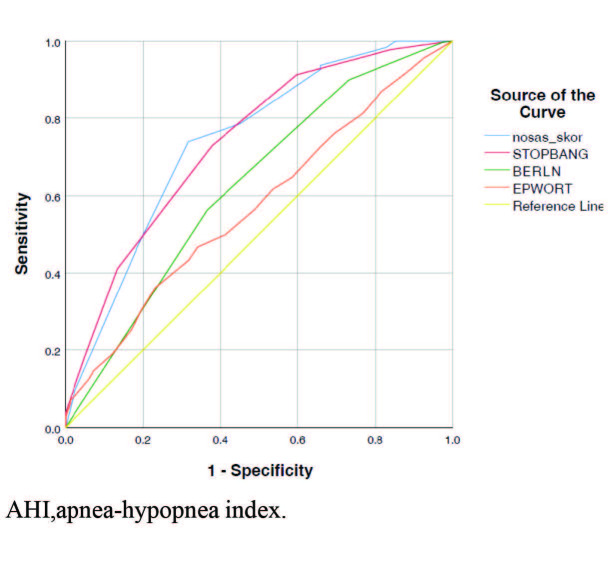
ROC curves of the 4 screening tools for an AHI ≥ 5 event/h. AHI: apnea–hypopnea index.

To compare the performance of the questionnaires for predicting SDB, ROC curves were constructed. We found that the AUC for the NoSAS score was larger than that of the 3 remaining questionnaires for all AHI cutoff points (Table 3). For an AHI ≥ 5 events/h, the NoSAS score had the largest AUC (0.740). It was followed by the STOP-Bang questionnaire (AUC: 0.737 for an AHI ≥ 5 event/h). The AUC of the ESS for an AHI ≥ 5 events/h was the lowest (Figure 2). For the other cut off points of AHI except an AHI ≥ 5 event/h, the Berlin score has the lowest AUC (0.569, 0.538, 0.539, 0.532, and 0.574 for AHIs ≥ 10, 15, 20, 25, 30, respectively).

**Table 3:** Performance of the NoSAS score compared with STOP-Bang, Berlin, and ESS scores (AUC).

Questionnaire	AHI≥5	AHI≥10	AHI≥15	AHI≥20	AHI≥25	AHI≥30
NoSAS	0.740	0.703	0.691	0.692	0.690	0.715
STOP-Bang	0.737	0.692	0.679	0.676	0.665	0.704
Berlin	0.626	0.569	0.538	0.539	0.532	0.574
ESS	0.571	0.576	0.600	0.587	0.607	0.621

Data are presented as values. AUC: area under the curve; AHI: apnea–hypopnea index; ESS: Epworth sleepiness score.

Overall, the NoSAS score correctly classified 298 of the 368 participants (81%) for an AHI ≥ 5 event/h and 223 of 264 participants (84.5%) for an AHI ≥ 15 event/h. The false negative rate for the NoSAS score was 9.1% for an AHI ≥ 15 event/h. When we compared the false negative group with the true positive group for NoSAS at the cut off point of AHI ≥ 15 event/h, there was a statistically significant difference regarding age, BMI, neck circumference, and for the male sex. In addition to this, significant differences were found between these 2 groups in terms of AHI and ODI (Table 4). The NoSAS score had a false negative rate of 2.9% for patients with severe SDB.

**Table 4:** Comparison of the false negative and the true positive patient groups for the NoSAS score.

	False negative group (n: 41)	True positive group (n: 223)	P value
Age, year	45.8 ± 7.9	53.1 ± 12.0	<0.001
BMI, kg/m2	29.2 ± 4.4	33.3 ± 6.30	<0.001
Neck circumference, cm	38.5 ± 1.5	42.5 ± 3.12	<0.001
Male sex (%)	53.7	77.6	0.001
Snoring (%)	95.1	97.3	0.453
AHI, event/h	31.1 ± 16.9	41.7 ± 22	0.002
ODI	47.7 ± 46.0	61.2 ± 46.7	<0.001

Data is depicted as mean ± SD or number (percentage). BMI: body mass index; AHI: apnea–hypopnea index; ODI: oxygen desaturation index.

The discriminative power of the NoSAS score was also evaluated considering differences in sex. The sensitivity of the NoSAS score was higher in male subjects than in those of female for AHI cut off points of 5, 15, and 30 events/h (87.4%, 88.7%, and 93.6%, respectively). However, specificity of the NoSAS score was better in females than in males for all AHI cut off points (Table 5). 

**Table 5:** Predictive parameters for the NoSAS score for SDB, considering differences in sex.

	Male			Female		
	AHI≥5	AHI≥15	AHI≥30	AHI≥5	AHI≥15	AHI≥30
Sensitivity(%)	87.4	88.7	93.6	65.1	72.5	81.8
Specificity(%)	28.9	21	19.4	70.5	60.5	53
PPV(%)	89.5	67.6	39.8	84.1	61	32.9
NPV(%)	25	50	84.1	45.6	72.1	91.2

SDB: sleep disordered breathing; PPV: positive predictive value; NPV: negative predictive value.

**Figure 3:**
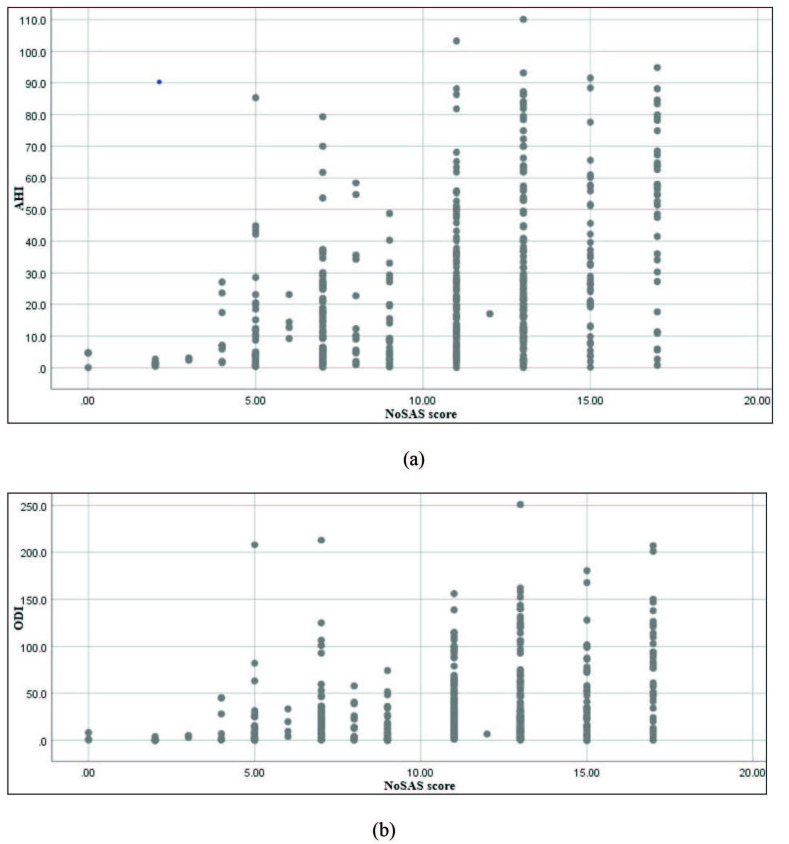
(A) Scatter plot figure with NoSAS and AHI; (B) Scatter plot figure with NoSAS and ODI. AHI: apnea–hypopnea index; ODI: oxygen desaturation index.

The Pearson correlation coefficient between NoSAS and STOP-Bang and NoSAS and Berlin was 0.714 and 0.316, respectively (P = 0.01). The correlation coefficient was not significant between NoSAS and ESS (r = 0.054; P > 0.05). Additionally, the NoSAS score was evaluated with AHI and ODI and described with scatter plot figures. (Figure 3A shows the scatter plot figure with NoSAS and AHI and Figure 3B shows the scatter plot figure with NoSAS and ODI).

## 4. Discussion

Sleep-disordered breathing is a major challenge for healthcare systems throughout the world [15]. To prevent the adverse outcomes of SDB, it is important to identify the high-risk patients for SDB [4]. Among the screening questionnaires developed for this purpose, the NoSAS score seems to be the most appropriate one for screening high-risk patients for SDB in a sleep clinical population. In this study, the NoSAS score demonstrated the largest AUC compared to the STOP-Bang, Berlin, and Epworth Sleepiness Scale for all cut off points of AHI. The NoSAS score is the best sensitivity–specificity compromise, allowing for the reductions in the number of unnecessary PSG recordings and the number of missed diagnoses of SDB. 

Using an AHI cut off of ≥20 event/h, we found that the sensitivity of the NoSAS score was higher (86%) than that reported in the HypnoLaus (79%), EPISONA (85%), Chinese (74.8%), and Asian (69.4%) cohorts [8,16,17]. In the HypnoLaus, EPISONA, and Chinese cohorts, it was discussed that the NoSAS score had a better performance than the STOP-Bang and Berlin questionnaires. Clinically significant SDB is defined as an AHI ≥ 20 event/h in these cohorts. In the Asian population, Tan A et al. found that the sensitivity of the NoSAS score was lower than that reported in the HypnoLaus and EPISONA cohorts and stated that the NoSAS score had a similar performance to the STOP-Bang and Berlin questionnaires [17]. In terms of specificity, our results represented the lowest levels compared to the HypnoLaus, EPISONA, Chinese, and Asian cohorts. However, when we compared the NoSAS score with the STOP-Bang and Berlin questionnaires in our population—although it is not perfect—it stands out due to its having a higher specificity than the other questionnaires (51.6%, 15,9%, and 26.8%, respectively for an AHI ≥ 5 event/h). The sensitivities of the STOP-Bang and the Berlin questionnaires were high in this study, but their specificities were rather unsatisfactory. The low specificity of the questionnaires may result in unnecessary sleep studies and have been documented in similar population samples [18,19]. The fact that the NoSAS score consists of objective parameters may also have played a role in its having a higher specificity than the other questionnaires in our population. Additionally, containing any subjective parameter except for snoring makes the NoSAS score more convenient for clinicians to use [20].  We also compared the discriminative power of the screening tools, and it was found that the diagnostic performance of the NoSAS score was better than the other 3 screening questionnaires. The AUC for an AHI ≥ 5 was 0.740, and this was the largest AUC of all AHI cut offs. The STOP-Bang questionnaire demonstrated a similar but lower AUC for an AHI ≥ 5 compared to the NoSAS score. In the Chinese cohort study performed in a sleep clinic population, the AUC of the Berlin questionnaire for an AHI ≥ 5 and AHI ≥ 10 was higher than that of NoSAS. However, the NoSAS score resulted in a better performance than the Berlin questionnaire for the other cut offs of AHI. In our study, the Berlin Questionnaire was the worst performing questionnaire in terms of AUC at an AHI ≥ 10 events/h. The Berlin questionnaire is complex and not entirely consistent with the actual situation in Turkey, for example when looking at questions related to falling asleep while driving. As reported in the study conducted by Peng et al., patients cannot answer the questionnaire if they don’t have driving experience, and this leads to the low feasibility of completion and poor accuracy in such scales. A previously mentioned study conducted in a sleep clinic population, the NoSAS score had the largest AUC (0.734) for an AHI ≥ 5 [20]. When studies in sleep clinic populations are evaluated, it can be concluded that the NoSAS score is a screening questionnaire that can be used not only for detecting moderate-to-severe SDB but also for detecting mild SDB in sleep clinic populations. Identification of mild SDB is as significant as moderate-to-severe SDB. Previous population studies have provided important information linking mild SDB with adverse cardiovascular and metabolic outcomes [21,22]. The sleep hearth health study also demonstrated a significant worsening of quality of life in subjects with mild SDB [23]. The accumulating literature on mild SDB provides clear evidence that mild SDB can be treated and that such a treatment can lead to an improvement in adverse health outcomes [24]. 

It is important not to neglect high-risk patients for SDB as well as to not perform unnecessary PSG recordings. In our study, for moderate SDB, the false negative rate of the NoSAS score was 9.1%, while it was 2.9% for severe SDB. The false negative rate for severe SDB was similar in the HypnoLaus cohort study. When the false negative group and the true positive group are compared in terms of the NoSAS score, it was observed that patients in the false negative group were weaker, younger, and had smaller neck circumferences; also, the number of male patients in the group was lower. It has been hypothesized that the sleep disordered breathing of the patients with a false negative result in the NoSAS score was more likely to be related to maxillofacial deformities, a high loop gain, or upper airway muscle control dysfunction than to obesity [8]. Care should be taken not to ignore this group of patients, and further examinations should be scheduled in the event of clinical suspicion. There is no significant difference between the 2 groups in terms of snoring, which is the only subjective variable of the NoSAS score. This suggests that objective criteria are more effective in forming the true positive group. The false negative group was made up of milder SDB patients than the true positive group, and the oxygen desaturation index was lower in this group. 

When the effects of differences in sex on the NoSAS score were evaluated, it was seen that the sensitivity of the NoSAS score was better in males than in females. However, specificity was better in females than in males. In other words, the NoSAS score was more successful in the roll-in in males and in the roll-out in females. The fact that the male sex is a variable of the NoSAS score may have affected this situation. However, sex as an indicator adds only 2 points to the overall score. In males, sensitivity was quite high in all SDB groups (mild, moderate, and severe), whereas specificity was low. In the study conducted by Mou J et al., STOP-Bang performance by sex showed extremely low specificity in males at the cutoff of ≥3, and alternative models were recommended [25]. Similarly, models can be developed to increase the specificity in males within the NoSAS score. In females, the NoSAS score, particularly for moderate to severe SDB, demonstrated good results in terms of both sensitivity and specificity. With increasing obesity rates worldwide, the incidence of SDB is increasing in women as well as in men. In a study conducted in Turkey, the incidence of high-risk SDB has been found to be higher in women than in men [26]. To our knowledge, this is the first study that evaluates the effects of differences in sex on the NoSAS score.

Our study also has some limitations. As one of the aims of this study was to validate the NoSAS score in a sleep clinical population, we retrospectively analyzed the value of the NoSAS score in our sleep center. The use of retrospective analysis to validate a screening tool is less ideal than a prospective study. However, this is an observational study. All of the data such as ESS, the Berlin and STOP-Bang scores, and biometric measurements were collected before the PSG recording was initiated. In addition, although our sleep center also provides services to patients from provinces other than İzmir, this was a single center study based on a specific Turkish population.

In conclusion, the NoSAS score performed better than the ESS and Berlin and STOP-Bang questionnaires in its identification of high-risk patients for SDB. The NoSAS score performed better than the other 3 screening tools, not only in moderate to severe SDB but also in mild SDB. It is an easily applicable, reliable, and subjective screening tool that can be effectively used in a sleep clinic population. 

## References

[ref1] (2013). Increased prevalence of sleep-disordered breathing in adults. American Journal of Epidemiology.

[ref2] (1997). Estimation of the clinically diagnosed proportion of sleep apnea syndrome in middle-aged men and women. Sleep.

[ref3] (2015). Prevalence of sleep-disordered breathing in the general population: the HypnoLaus study. Lancet Respiratory Medicine.

[ref4] (2004). Access to diagnosis and treatment of patients with suspected sleep apnea. American Journal of Respiratory and Critical Care Medicine.

[ref5] (2018). Screening questionnaires for obstructive sleep apnea: an updated systematic review. Oman Medical Journal.

[ref6] (2017). Diagnostic accuracy of the Berlin questionnaire, STOP-BANG, STOP, and Epworth sleepiness scale in detecting obstructive sleep apnea: A bivariate meta-analysis. Sleep Medicine Reviews.

[ref7] (1991). A new method for measuring daytime sleepiness: the Epworth sleepiness scale. Sleep.

[ref8] (2016). The NoSAS score for screening of sleep-disordered breathing: a derivation and validation study. Lancet Respiratory Medicine.

[ref9] (1999). Using the Berlin questionnaire to identify patients at risk for the sleep apnea syndrome. Annals of Internal Medicine.

[ref10] (2008). Vairavanathan S et al. Anesthesiology.

[ref11] (2008). Reliability and validity studies of the Turkish version of the Epworth Sleepiness scale. Sleep and Breathing.

[ref12] (2015). The effect of adding gender item to Berlin questionnaire in determining obstructive sleep apnea in sleep clinics. Annals of Thoracic Medicine.

[ref13] (2013). Günal Eruyar S et al. Turkish Journal of Anaesthesiology and Reanimation.

[ref14] (2016). The AASM Manual for the Scoring of Sleep and Associated Events: Rules, Terminology. Technical Specifications, Version.

[ref15] (2018). Challenges and perspectives in obstructive sleep apnoea: report by an ad hoc working group of the sleep disordered breathing group of the European Respiratory Society and the European Sleep Research Society. Respiratory Journal.

[ref16] (2018). Validation of the NoSAS Score for the screening of sleep-disordered breathing: a hospital-based retrospective study in China. Journal of Clinical Sleep Medicine.

[ref17] (2017). Validation of NoSAS score for screening of sleep-disordered breathing in a multiethnic Asian population. Sleep and Breathing.

[ref18] (2016). Weighted STOP-Bang and screening for sleep-disordered breathing. Sleep and Breathing.

[ref19] (2011). Identification of patients with sleep disordered breathing: comparing the four-variable screening tool, STOP, STOP-Bang, and Epworth sleepiness scales. Journal of Clinical Sleep Medicine.

[ref20] (2018). Application value of the NoSAS score for screening sleep-disordered breathing. Journal of Thoracic Disease.

[ref21] (2000). Prospective study of the association between sleep-disordered breathing and hypertension. New England Journal of Medicine.

[ref22] (2001). Sleep-disordered breathing and cardiovascular disease: cross-sectional results of the sleep heart health study. American Journal of Respiratory and Critical Care Medicine.

[ref23] (2001). The association of sleep-disordered breathing and sleep symptoms with quality of life in the Sleep Heart Health Study. Sleep.

[ref24] (2007). Mild obstructive sleep apnea syndrome should be treated. Journal of Clinical Sleep Medicine.

[ref25] (2018). The discriminative power of STOP-Bang as a screening tool for suspected obstructive sleep apnea in clinically referred patients: considering gender differences. Sleep and Breathing.

[ref26] (2012). Prevalence and associated factors of sleep-disordered breathing in the Turkish adult population. Sleep and Biological Rhythms.

